# Role of Non-pharmacological Interventions and Weight Loss in the Management of Gastroesophageal Reflux Disease in Obese Individuals: A Systematic Review

**DOI:** 10.7759/cureus.28637

**Published:** 2022-08-31

**Authors:** Maria Mukhtar, Mohammed J Alzubaidee, Raga Sruthi Dwarampudi, Sheena Mathew, Sumahitha Bichenapally, Vahe Khachatryan, Asmaa Muazzam, Chandani Hamal, Lakshmi Sai Deepak Reddy Velugoti, Godfrey Tabowei, Greeshma N Gaddipati, Safeera Khan

**Affiliations:** 1 Research, California Institute of Behavioral Neurosciences & Psychology, Fairfield, USA; 2 Internal Medicine, California Institute of Behavioral Neurosciences & Psychology, Fairfield, USA; 3 Pathology Research, California Institute of Behavioral Neurosciences & Psychology, Fairfield, USA

**Keywords:** obesity and weight loss and gerd, weight loss and gerd, obesity and gerd, gerd, obesity

## Abstract

With the increasing prevalence of obesity, the worldwide risk of gastroesophageal reflux disease (GERD) has also increased. Abdominal obesity increases intragastric pressure, disturbing the integrity of the gastroesophageal junction, thus facilitating reflux. Other than obesity, some lifestyle factors also cause GERD, including smoking, consumption of alcohol and caffeine, late-night meals, and high fat intake. This review study aimed to assess the impact of weight loss and lifestyle modifications on GERD. In this systematic review, the databases used were PubMed, PubMed Central (PMC), Science Direct, and Google Scholar. Boolean system and Medical Subject Headings (MeSH) strategy were used to form suitable keywords. Patients from the pediatric and geriatric populations were excluded from the study and quality assessment was done using different assessment tools. A positive association between obesity and GERD was found. It was also found that the long-term use of proton pump inhibitors (PPIs) causes complications, so lifestyle interventions should be used more than PPIs for treating GERD, especially in obese patients. We concluded that weight loss could lead to the resolution of gastroesophageal reflux disease, and therefore, conservative measures, including dietary modifications such as reducing the consumption of alcohol, caffeine, and chocolate, behavioral changes such as smoking cessation and elevation of the head of the bed, and weight loss, should be used as first-line management for GERD. Although awareness has increased regarding the adverse effects of proton pump inhibitors, future studies are required to assess these negative effects.

## Introduction and background

Various epidemiological studies have shown that the prevalence of gastroesophageal reflux disease (GERD) is increasing worldwide, and the major contributing factor to this trend is the rising prevalence of obesity. The worldwide estimated prevalence of GERD ranges from 15% to 25%. Western countries, including the United State of America, have a GERD prevalence higher than in Asia, ranging from 10% to 30% [1,2]. Other than obesity, several environmental and lifestyle factors also contribute to this increasing trend, such as being overweight, smoking, and consumption of alcohol, caffeine, fat, and chocolate (these factors indirectly lead to obesity and overweight) [3]. In addition, estrogen also has an important role in the development of GERD; one study suggested that pre-menopausal and women on hormone replacement therapy are noticed to have a higher incidence of GERD symptoms [4]. Lifestyle modifications, including weight loss, elevation of the head of bed, smoking cessation, and avoiding late evening meals, lead to the resolution of GERD symptoms [5]. One of the previous cohort studies concluded that, in obese individuals, having a low-carbohydrate diet results in the improvement of reflux symptoms [6].

Most of the studies have found a positive correlation between obesity and GERD. Obesity has been defined as having BMI >30 kg/m^2^ in most of these studies. Four previous cross-sectional studies confirmed a positive association between overweight or obesity and GERD symptoms in the US, UK, Norwegian, and Spanish populations. Two studies have shown a dose-response relationship [7]. The primary mechanism by which obesity promotes GERD is unclear; recent data suggests that obesity increases the intragastric pressure causing relaxation of the lower esophageal sphincter (LES) and reflux of gastroduodenal contents causing the symptoms of heartburn, acid regurgitation, and eventually causing erosive esophagitis [8]. Erosive esophagitis eventually causes Barrett's esophagus, thus increasing the risk of esophageal adenocarcinoma. Treatment of GERD includes conservative and medical therapies. Recent studies suggested that the long-term use of and proton pump inhibitors (PPIs) can cause several adverse effects.

A retrospective cohort study conducted by Bang and Park in 2018 concluded a positive association between a higher BMI and the development of GERD and erosive esophagitis. Conversely, this study also suggested that a decrease in the BMI can lead to the resolution of erosive esophagitis, and weight loss is a potentially effective treatment of GERD [3]. Furthermore, it is noticed that in obese individuals, asymptomatic GERD is more common than symptomatic reflux disease [9].

Although many studies have shown that GERD is associated with obesity, not all studies have shown a positive association. For example, two extensive population-based studies from Sweden and Denmark found no association. Researchers assumed that the disparity in results might be due to the non-adjustment of confounding variables [7]. In addition, weight loss had an independent effect on reflux symptoms in an obese individual. Still, we couldn't find sufficient data to suggest the positive impact of weight loss achieved through different methods, either with lifestyle modification or with surgical procedures (Roux-en-Y gastric bypass or vertical band gastroplasty), on GERD symptoms [10]. This systematic review aims to assess non-pharmacological interventions for GERD treatment and the impact of weight loss on GERD in obese patients.

## Review

Methodology

Study Design and Search Strategy

This systematic review was designed according to the Preferred Reporting Items for Systematic Reviews and Meta-Analyses (PRISMA) guidelines to improve reporting of this review [11]. We thoroughly searched through the following databases: PubMed, PubMed Central (PMC), Science Direct, and Google Scholar, using suitable keywords and Medical Subject Headings (MeSH) terms to extract all the relevant articles. We used the Boolean scheme and MeSH strategy to form keywords. The MeSH strategy used was (“Obesity/diet therapy"[Mesh] OR “Obesity/drug therapy"[Mesh] OR “Obesity/prevention and control"[Mesh]) AND (“Gastroesophageal Reflux/diet therapy"[Majr] OR “Gastroesophageal Reflux/drug therapy"[Majr] OR “Gastroesophageal Reflux/prevention and control"[Majr] OR “Gastroesophageal Reflux/therapy"[Majr]). For other databases, we used the following keywords: Obesity, Obesity AND GERD, Weight loss AND GERD, Obesity AND weight loss AND GERD. We removed duplicates by carefully scrutinizing the titles, and subsequently, we excluded irrelevant articles by screening the titles and abstract.

Inclusion and Exclusion Criteria

In this review, we included articles published in the English language, focusing on the adult population (18-65 years) and papers relevant to the research question. We excluded papers focusing on pediatric and geriatric populations and unpublished and grey literature.

Data Extraction

After we did the quality assessment, data extraction from the eligible papers included in the study was done. Two researchers did data selection and extraction (first and second authors). We reviewed the study design, relevance to our inclusion and exclusion criteria, intervention used, and outcomes measured in the selected paper.

Quality Assessment of the Studies

We used the Joanna Briggs Institute (JBI) critical appraisal checklist for cross-sectional studies and the Newcastle-Ottawa assessment tool for other observational studies (case-control, cohort). In addition, we used the assessment of multiple systematic reviews (AMSTAR) tool for the quality appraisal of the systematic reviews.

Results

**Figure 1:**
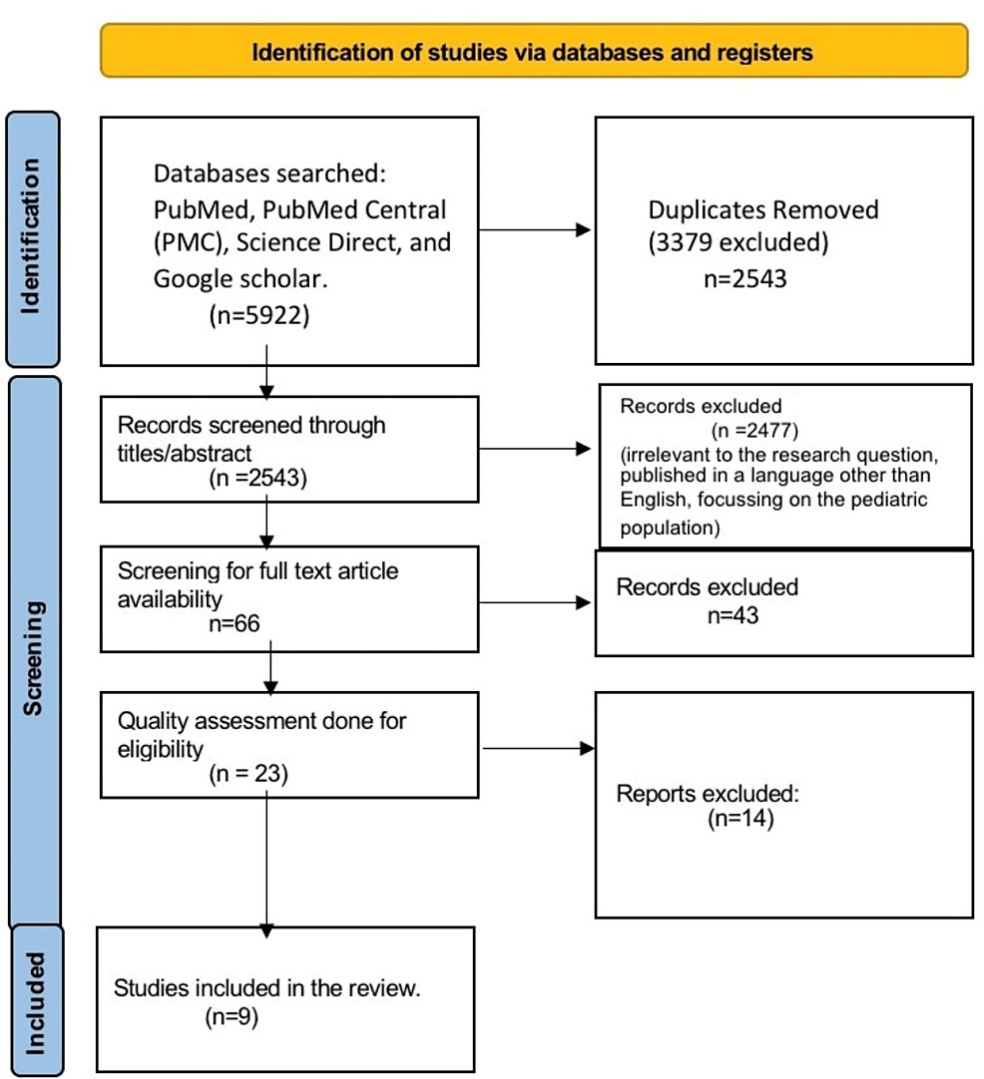
PRISMA flow chart PRISMA, Preferred Reporting Items for Systematic Reviews and Meta-Analyses

A total of 5922 papers were found after the database search. After removing 3379 duplicates, 2543 articles remained. Then screening was done through titles, and we removed 2477 articles because of irrelevance, leaving 66 articles. Next, we checked the availability of full-text articles and pulled 43 out of 66 because of the unavailability of full-text articles, and a total of 23 articles remained. Then critical appraisal was done using different quality assessment tools, and a total of eight articles were found eligible to be included in our study after quality assessment. Out of eight papers, five were cross-sectional studies, three cohort studies, and one was a systematic review. The complete PRISMA flow diagram is well explained in Figure 1 [11].

We set the cut-off for bias at 20% or less to be included in this study (Table 1).

**Table 1:** Risk of bias assessment GERD, gastroesophageal reflux disease; JBI, Joanna Briggs Institute; AMSTAR, assessment of multiple systematic reviews

Authors	Study title	Study type	Assessment tool used	Score	Risk of bias
Bang and Park et al. [3]	Weight loss as a nonpharmacologic strategy for erosive esophagitis: a 5-year follow-up study	Retrospective cohort study	Newcastle-Ottawa tool	8 out of 9	10%
Ness-Jensen et al. [5]	Lifestyle intervention in gastroesophageal reflux disease	Systematic review	AMSTAR	Moderate quality review	Low risk
Hashem et al. [7]	Obesity is an independent risk factor for GERD symptoms and erosive esophagitis	Cross-sectional	JBI tool	6 out of 8	20%
Singh et al. [10]	Weight loss can lead to resolution of gastroesophageal reflux disease symptoms	Prospective cohort study	Newcastle-Ottawa tool	7 out of 9	20%
Fraser-Moodie et al. [12]	Weight loss has an independent beneficial effect on symptoms of gastro-esophageal reflux in patients who are overweight	Prospective cohort study	Newcastle-Ottawa tool	7 out of 9	20%
Aslam et al. [13]	Nonlinear relationship between body mass index and esophageal acid exposure in the extraesophageal manifestations of reflux	Cross-sectional	JBI tool	6 out of 8	20%
Wu et al. [14]	Obesity Is associated with increased transient lower esophageal sphincter relaxation	Cross-sectional	JBI tool	6 out of 8	20%
Nocon et al. [15]	Association of body mass index with heartburn, regurgitation and esophagitis: results of the Progression of Gastroesophageal Reflux Disease study	Cross-sectional	JBI tool	6 out of 8	20%

Out of five cross-sectional studies, four concluded that obese and overweight individuals have an increased risk of GERD. Obesity causes disintegration of LES and reflux of acidic gastric content in the esophagus, increasing the risk of erosive esophagitis. One cross-sectional study investigated the benefits of weight loss on GERD symptoms. All three cohort studies reported that weight loss could lead to the resolution of GERD and erosive esophagitis in obese patients. One systematic review investigated the impact of lifestyle intervention on GERD symptoms. That review also reported several adverse effects of the long-term use of proton pump inhibitors. The study concluded that because of some complications and the high cost of PPIs, lifestyle interventions, especially weight loss, should be used as first-line management for GERD in obese individuals.

Discussion

The reflux of gastroduodenal contents from the stomach into the esophagus due to any anatomical defect at the gastroesophageal junction (GEJ) is defined as gastroesophageal reflux disease [12]. Over the last few decades, the prevalence of GERD has been increasing worldwide. Most of studies have found that this increase is due to the increase in the prevalence of obesity worldwide [10]. Obesity is an independent risk factor for developing GERD symptoms, and several studies have been conducted to find a positive association between BMI and GERD. Jacobson et al. conducted a case-control study in 2006 and found that BMI is directly associated with GERD irrespective of whether the individual is with normal weight or overweight [16]. Obesity has been associated with various comorbidities including diabetes mellitus and cardiovascular diseases; however, gastrointestinal disorders including GERD, gall stones and non-alcoholic fatty liver disease (NAFLD) are more frequent in obese individuals [17,18]. Milić et al. conducted a study in 2014 and concluded that NAFLD is associated with obesity and the main treatment option for NAFLD would be weight reduction and life style modification [19].

Pathophysiology

The intra-abdominal pressure theory states that obesity in general and abdominal obesity increase intragastric pressure that causes mechanical stress on the gastroesophageal junction, causing transient lower esophageal sphincter relaxation (TLOSR) and predisposition of the hiatus hernia, which further facilitates reflux. The dietary habit theory suggests that some nutritional habits may be the main reason for increasing the risk of obesity and eventually increasing GERD risk. Both approaches are based on assumptions [13]. Hiatus hernia in obese individuals causes disturbances in the integrity of GEJ, thus aggravating reflux [20]. Obesity may cause humoral changes such as changes in leptin and insulin levels and hormonal changes such as changes in estrogen levels, which indirectly cause GERD [7]. Not enough evidence supports these mechanisms, so further studies are required to explore them.

A study conducted by Wu et al. in 2007 concluded that abnormal relaxation of the lower esophageal sphincter (LOS) might cause obesity-related GERD [14]. In this study, patients were divided into three groups: obese (BMI >30 kg/m^2^), overweight (BMI 25-30), and average weight (BMI <25), according to the World Health Organization (WHO). During the postprandial period, esophageal manometry and pH monitoring were done using the standard method. It was noted that TLOSR rates were higher in obese and overweight subjects. It was speculated that obese patients tend to overeat, causing an increase in intragastric pressure and gastric distention, disrupting the integrity of the LOS and causing acid reflux. Esophageal manometry was done in obese individuals before bariatric surgery and most of the people were noted to have motility disorder; this could be another reason for GERD in overweight individuals [21].

Non-pharmacological Interventions

Although obesity is a significant independent risk factor for GERD, several other factors are also noted to play an essential role in GERD development. In a recent retrospective cohort study conducted by Bang and Park, it was stated that GERD is associated with some dietary habits, for example, late-night meals, consumption of alcohol, caffeine, chocolate, fat, and smoking [3]. The intake of citrus fruit is also noted to aggravate the GERD symptoms [22]. Non-pharmacological interventions, including smoking cessation, avoiding late-night meals, reducing the consumption of alcohol and caffeine, and behavioral changes such as weight loss, can lead to the resolution of GERD symptoms [3,5]. Another study suggested that elevating the head of the bed in the supine position and lying on the left side helps to improve reflux symptoms [23]. Furthermore, regular physical activity was noted to have a positive effect on GERD symptoms [24,25].

Smoking Cessation

Most studies have shown a positive association between smoking and GERD symptoms. In a recent systematic review conducted by Ness-Jensen et al., it was revealed that smoking reduces the LOS pressure facilitating reflux [5]. Also, it causes decreased secretion of salivary bicarbonate, eventually decreasing acid buffering. Another study concluded that smoking cessation was associated with decreased reflux symptoms in normal weight individuals. However, in obese individuals, obesity was the leading cause of GERD, so smoking cessation didn't help much in that group [5].

Lifestyle Modification

Some studies showed a positive association between some dietary habits, lifestyle factors, and the development of GERD symptoms, but previous data supporting these facts are scarce. For example, a randomized controlled trial (RCT) conducted with a small sample size (only 15 patients) showed that the elevation of the head of the bed decreased the time for which lower esophageal pH was <4 [5]. Another RCT demonstrated that an increased dietary fat intake causes more time without heartburn symptoms. A systematic review has shown that a high fiber intake and moderate physical activity can reduce GERD symptoms [5]. A recent cohort study suggested that adjusting meal size and timing, i.e., avoiding late-night meals, is reported to be helpful for the management of GERD [3]. Although physical activity helps to improve the symptoms, but vigorous exercise after a meal can worsen the condition. Post-dinner walking is recommended to relieve the reflux and heartburn, but eating before exercise should be avoided [26].

Weight Loss as a Management of GERD

Many observational and experimental studies confirmed the association between a high BMI and GERD. A retrospective cohort study was conducted in 2018 to investigate whether a decreased BMI can resolve erosive esophagitis (EE) [3]. All the participants underwent upper GI endoscopy and EE was classified according to Los Angeles (LA) classification; the baseline BMI was noted and all patients were instructed to lose weight. During five-year follow-up periods, the EE resolution rate was higher in subjects who had a decrease in BMI >2 kg/m^2^; the researchers concluded that a significant weight loss is required for EE resolution because some of the patients who had a reduction in BMI <1 kg/m^2^ did not show any positive effects [3]. Weight loss reduces intragastric pressure and pressure on the gastroesophageal junction, thus reducing the reflux episodes [10]. An RCT of 17 patients reported normalization of the esophageal pH with weight loss in a follow-up period of four months.

A cross-sectional study conducted in 2006 suggested that weight loss is an effective treatment for GERD. Still, two other studies showed contradictory results, stating that a reduction in the BMI does not cause improvement in healing rates after proton pump inhibitor use [15]. Other than heartburn and acid regurgitation, some extraesophageal manifestations of GERD include cough, hoarseness, asthma, sore throat, sinusitis, and globus sensation. There is a significant and nonlinear relationship between a higher BMI and GERD with extraesophageal manifestations reported in a previous study conducted by Aslam et al. The study concluded that an increased BMI is significantly associated with esophageal acid exposure and these findings suggest the benefit of weight loss in the treatment of GERD [13]. Fraser-Moodie et al. conducted a prospective cohort study in 2014 to assess an independent effect of weight loss on the improvement of GERD symptoms [12]. This study found that weight loss is the first line of management in treating GERD in overweight patients. Weight loss either achieved through lifestyle interventions or through bariatric surgery was noticed to have improvement in symptoms of GERD [27].

Individuals can use different strategies to lose weight, including physical activity, dietary modifications, and behavioral changes. Physical activity can include walking or some other exercises. In a prospective cohort study, overweight patients were followed for six months for weight loss. Weight loss was achieved through different conservative measures, such as increasing physical activity, some dietary modifications that reduced the daily calorie intake to 1200-1500 cal/day, and some behavioral changes, including goal-setting, self-monitoring, feedback, reinforcement, and social support. Most patients lost weight, and with a structured weight loss program, GERD symptoms were entirely resolved. In addition, a dose-response relationship was reported between weight loss and the resolution of GERD symptoms [10]. But not all obese patients in this study had a reduction in GERD symptoms after losing weight [5].

Why Is Weight Loss More Effective Than PPIs for GERD Symptom Resolution in Obese Patients?

Treatment options for gastroesophageal reflux disease include conservative measures and medical treatment. Conservative measures, as already mentioned, include weight loss, if the patient is obese and overweight, the elevation of the bed head, avoiding late-night meals, and reducing the consumption of alcohol, fat, caffeine, and chocolate intake [3,5]. Medical treatment is commonly achieved through antacids, H2 receptor antagonists (H2RAs), and proton pump inhibitors for heartburn and acid regurgitation [28]. PPIs work by inhibiting the acid secretion from parietal cells. Additionally, if symptoms are non-responsive to medical treatment or complications have developed, then surgical treatment is also recommended especially in the presence of hiatal hernia [29].

Initially, PPIs were mainly used for treating GERD symptoms, but recently, some studies suggested that the long-term use of proton pump inhibitors can cause some adverse effects. Now that awareness about the side effects of PPIs has increased, lifestyle modification is preferred. For example, withdrawal of PPIs induces reflux symptoms. Other adverse effects include hypergastrinemia and rebound acid secretion. In addition, due to increased gastric pH, the risk of enteric infection and community-acquired pneumonia is increased. There is also an increased risk of hip fractures because of malabsorption of calcium [5].

Another study suggested that obese individuals require a long-term use of H2RAs and antacids for heartburn and reflux symptoms, concluding that obese individual are not as responsive to medications for GERD. Lifestyle interventions, including weight loss and smoking cessation, having a low economic cost and no harmful side effects, should be preferred for GERD treatment. Although proton pump inhibitors positively impact the resolution of GERD symptoms and extensive data supports these positive results, very few RCTs and observational studies are available investigating the positive effects of lifestyle interventions on GERD.

Weight loss should be used as the first-line treatment for GERD in obese and overweight patients because of its low-cost effects, preventing complications of GERD, and improving the quality of life. It was estimated in a recent study that around 10 billion US dollars a year are spent on medical treatment of GERD, and that is considered a burden on the healthcare system [10]. However, there are a few limitations of our study. Extensive data is available reporting the positive association between obesity and GERD development, but studies suggesting the impact of weight loss on GERD are scarce. We could not find sufficient RCTs and observational studies suggesting that weight loss is an effective treatment for GERD.

Further studies, including clinical trials for evaluating the effect of weight loss on symptoms of reflux, are required. Two extensive studies reporting the impact of weight loss on GERD produced contradictory conclusions [15]. Another limitation is that our research does not apply to the pediatric and geriatric population; it is only limited to the adult population (18-65 years).

## Conclusions

We conducted this review to assess the impact of weight loss and lifestyle modification on the symptoms of gastroesophageal reflux disease in obese and overweight individuals. Obesity is an independent risk factor for GERD; abdominal obesity increases intragastric pressure and transient relaxation of the lower esophageal sphincter facilitating the reflux. Weight loss reduces the mechanical stress on the gastroesophageal junction, decreasing the reflux and leading to GERD resolution. The long-term use of proton pump inhibitors can cause several adverse effects, and also, it is an economic burden on the healthcare system. Lifestyle modifications, including smoking cessation, avoiding late-night meals, reducing the consumption of alcohol, caffeine, and chocolate, and increasing the fiber intake in the diet, are reported to be useful measures in gastroesophageal reflux disease. Because of cost-effectiveness and no adverse effects, conservative measures should be used as the first-line treatment for the management of heartburn and acid regurgitation symptoms. Further studies, including clinical trials, should report the adverse effects of PPIs and the benefits of using conservative measures, especially in obese individuals with gastroesophageal reflux disease.
